# A Prospective Assessment of Hearing Threshold Variability in Personal Listening Device Users

**DOI:** 10.7759/cureus.110339

**Published:** 2026-06-06

**Authors:** Bhushan Chauhan, Shweta Godara, Aishwarya Jayan, Kirtika Gupta, Ginni Datta

**Affiliations:** 1 Department of ENT, Maharishi Markandeshwar (MM) Institute of Medical Science and Research, Ambala, IND

**Keywords:** earphone usage, hearing threshold shift, noise-induced hearing loss, personal listening devices, volume intensity, young adults

## Abstract

Background and aim

The increasing use of personal listening devices (PLDs), particularly in-ear earphones, has raised concerns regarding their potential adverse effects on auditory health among young adults. Prolonged exposure to high-intensity recreational sound is considered a major risk factor for noise-induced hearing loss (NIHL). However, longitudinal evidence evaluating the combined influence of listening duration and volume intensity on hearing thresholds remains limited. This study aimed to prospectively assess hearing threshold variability among PLD users and examine the dose-dependent effects of earphone usage duration and listening volume over an 18-month period.

Materials and methods

A prospective longitudinal study was conducted among 100 participants aged 18-25 years over 18 months. Participants were categorized into four groups (n=25 each) based on daily earphone usage duration as follows: group A (1-2 h/day), group B (2-4 h/day), group C (>4 h/day), and group D (≤1 h/day/non-users; control). Each group was further stratified according to listening volume intensity (<60%, 60-80%, and >80% based on device volume settings). Baseline demographic details and otology symptoms were recorded using a structured questionnaire. Audiological evaluation was performed using pure-tone audiometry (PTA) at baseline and at 6, 12, and 18 months under standardized conditions. Statistical analysis was conducted using ANOVA and post hoc Tukey tests, with p≤0.05 considered statistically significant.

Results

A progressive increase in mean hearing thresholds was observed among earphone users over the 18-month follow-up period, with the highest deterioration noted in group C. Mean hearing thresholds increased from 13.36±3.90 dB to 14.92±4.41 dB in group A, from 15.42±4.69 dB to 18.42±4.86 dB in group B, and from 15.02±5.40 dB to 20.30±6.01 dB in group C, whereas minimal variation was observed in the control group (8.58±2.07 dB to 8.86±2.07 dB). Hearing threshold shifts demonstrated a dose-dependent pattern, with the highest shift observed in group C (-5.28±1.73 dB), followed by group B (-3.00±0.95 dB) and group A (-1.56±1.05 dB). Higher listening volume (>80%) was significantly associated with greater auditory decline and increased prevalence of otology symptoms such as tinnitus and ear pain. Significant intergroup differences were observed (p=0.001).

Conclusion

Prolonged and high-volume use of in-ear earphones was associated with progressive hearing threshold shifts among young adults, suggesting early auditory changes consistent with recreational noise exposure. Both listening duration and volume intensity emerged as important modifiable risk factors. The findings highlight the importance of safe listening practices, early auditory screening, and public health awareness strategies to reduce the potential risk of NIHL associated with personal listening device usage.

## Introduction

Personal listening devices (PLDs), particularly in-ear earphones, have become an integral part of daily life, especially among adolescents and young adults, offering convenience, portability, and personalized audio experiences for communication, education, and entertainment. Notably, more than 1.1 billion adolescents and young adults aged between 12 and 35 years are at increased risk of developing hearing loss and permanent auditory damage at later stages of life owing to prolonged use of PLDs and frequent exposure to loud noise entertainment [1,2]. Unlike occupational noise exposure, recreational listening through PLDs is predominantly self-regulated, with limited awareness of safe listening practices, thereby increasing the risk of excessive sound exposure. However, such behaviors are potentially modifiable through effective public health interventions and implementation of established policy recommendations [3].

The design of in-ear earphones allows sound to be delivered directly into the external auditory canal, which can amplify the intensity of sound at or on the tympanic membrane. Prolonged listening at high volume, particularly above 85 dB and between 3 and 8 kHz, can cause irreversible damage to cochlear hair cells, leading to noise-induced hearing loss (NIHL) [4]. NIHL develops gradually and often remains subclinical during early stages, making timely diagnosis difficult until significant hearing loss or auditory impairment occurs. Early symptoms may include tinnitus, difficulty in understanding speech in noisy environments, and increased listening fatigue [5,6].

The recurrent exposure to unsafe listening not only contributes to transient or permanent physiological damage to the auditory system but also affects academic performance, reduced concentration and motivation, social withdrawal, anxiety, depression, and poorer quality of life, especially among children. In adults, hearing impairment has been linked to adverse psychosocial outcomes, reduced economic productivity, cognitive decline, and other comorbid health conditions [1,2]. Despite these significant consequences, awareness and adoption of safe listening practices remain limited. Existing literature has limited evidence regarding the correlation between prolonged usage of PLDs at high volume and intensity and alterations in hearing thresholds. Further, the majority of studies are cross-sectional in nature, limiting the ability to assess correlations and cumulative exposure effects. Moreover, there is a notable lack of longitudinal research, particularly in developing countries, where cultural practices, lifestyle patterns, awareness levels, and prolonged duration of PLDs may influence listening behaviors and auditory outcomes differently [7,8]. This highlights the need for context-specific, long-term investigations to understand the impact of PLDs on auditory health.

Thus, the present prospective longitudinal study was designed to evaluate progressive hearing changes associated with in-ear earphone use among young adults aged 18-25 years over an 18-month period. The study specifically investigated the effects of earphone usage duration and volume intensity on hearing thresholds and auditory health.

## Materials and methods

A prospective longitudinal study was conducted in the department of otorhinolaryngology and head and neck surgery at a tertiary care hospital over a period of 18 months, including an initial two-week screening and recruitment phase followed by serial audiological assessments. This study aimed to evaluate the effect of prolonged in-ear earphone usage on hearing thresholds among young adults.

Participants aged 18-25 years were recruited through random sampling from students and young adults attending the outpatient department and institutional campus. A structured questionnaire was administered to obtain demographic information, patterns of personal listening device usage, preferred listening duration and volume settings, and associated otology symptoms such as tinnitus, ear pain, and subjective hearing difficulty.

Control of confounding variables

To minimize confounding factors, participants with significant exposure to environmental or recreational noise, frequent gaming with headphones, or regular use of multiple high-volume audio devices were screened and, where applicable, excluded. Data on occupational noise exposure, residential noise environment, and use of additional audio devices were also collected via questionnaires. The schematic representation of the study and structured questionnaire are provided in supplementary data (appendix).

Inclusion and exclusion criteria

Participants using in-ear earphones for more than 1 h daily for a duration exceeding one month were included in the test groups. Subjects were categorized into the following three groups based on daily duration of earphone usage: group A (1-2 h/day), group B (2-4 h/day), and group C (>4 h/day). Individuals who did not use earphones or used them for less than 1 h daily at low volume were included in the control group (group D). Participants with pre-existing ear disorders, history of hearing impairment, use of ototoxic medications, chronic middle ear disease, prior ear surgery, or regular exposure to occupational/recreational loud noise sources such as industrial equipment, concerts, discos, music bands, firearm use, or shooting sports were excluded from the study to reduce confounding bias.

Sample size calculation

Sample size was calculated using G*Power software version. 3.1.9.7 (Düsseldorf, Germany: Heinrich Heine University Düsseldorf) based on an alpha error of 0.05 and statistical power of 90% (β=0.1). The minimum required sample size was estimated, after which 100 participants who met the inclusion criteria were enrolled and equally distributed into four groups (n=25 each).

Assessment of volume intensity

Participants in groups A, B, and C were further subdivided according to listening volume intensity into low (<60%), moderate (60-80%), and high (>80%) categories based on smartphone/device volume settings routinely used by the participants during earphone listening. These categories represented percentage-based volume output settings of personal listening devices rather than absolute sound intensity measured in decibels.

Audiological assessment

All eligible participants underwent comprehensive otorhinolaryngological examination followed by audiological evaluation at baseline, six months, 12 months, and 18 months. Hearing threshold shift (HTS) was defined as an increase in the minimum sound intensity, measured in decibels (dB), required for auditory perception relative to baseline hearing thresholds.

Pure-tone audiometry (PTA) was performed in a soundproof room, with ambient noise maintained below 35 dB, using a calibrated AC40 audiometer (Middelfart, Denmark: Interacoustics) and TDH-39 supra-aural headphones (Farmingdale, NY: Telephonics Corporation) in accordance with IEC 60645-1 standards. Air conduction thresholds were assessed between 0.125-8 kHz and bone conduction thresholds between 0.5-4 kHz. The testing procedures were conducted by trained audiology personnel under standardized conditions to ensure reliability and reproducibility across all follow-up assessments.

Statistical analysis

Statistical analysis was performed using SPSS version 21.0 (Chicago, IL: SPSS Inc.). Quantitative variables were expressed as mean±standard deviation (SD). Intergroup comparisons were performed using analysis of variance (ANOVA) followed by post hoc Tukey testing. Statistical significance was considered at p≤0.05.

## Results

The prospective study was conducted to evaluate the effect of usage of earphones on hearing thresholds among young adults. A total of 100 participants aged 18-25 years were enrolled and categorized into four groups with 25 participants in each group based on their average daily duration of earphone usage. Group A comprised participants who used earphones for 1-2 h per day, group B comprised participants who used earphones for 2-4 h per day, and group C comprised participants who used earphones for more than 4 h per day. Group D served as the control group and included the participants who either did not use earphones or used them for less than 1 h per day. Audiometric evaluations were conducted periodically at 0, six, 12, and 18 months to monitor hearing threshold changes over time. The data were analyzed to compare hearing threshold changes in terms of duration and volume intensity with earphone usage.

Age distribution

**Figure 1:**
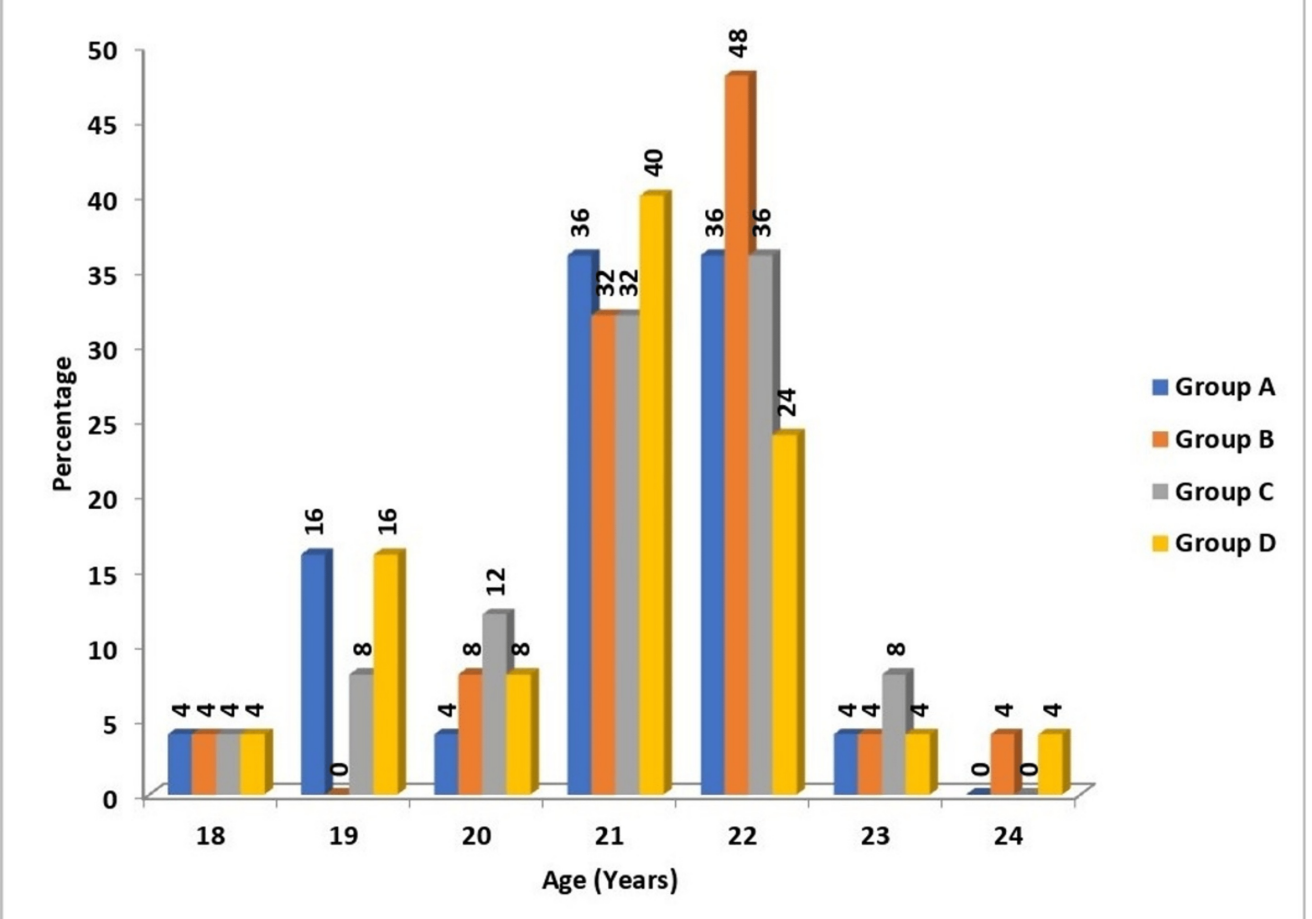
Age wise distribution of participants.

The age distribution of participants is presented in Figure 1, reflecting higher usage of earphones in the early 20s. The majority of participants were within 21 (35%) and 22 (36%) years, with group A showing equal number of participants (36%). Group B included 32% and 48% participants aged 21 and 22 years, respectively. Group C had eight (32%) participants aged 21 years and nine (36%) aged 22 years, while group D included 10 (40%) participants aged 21 years and six (24%) aged 22 years.

Gender distribution

**Figure 2:**
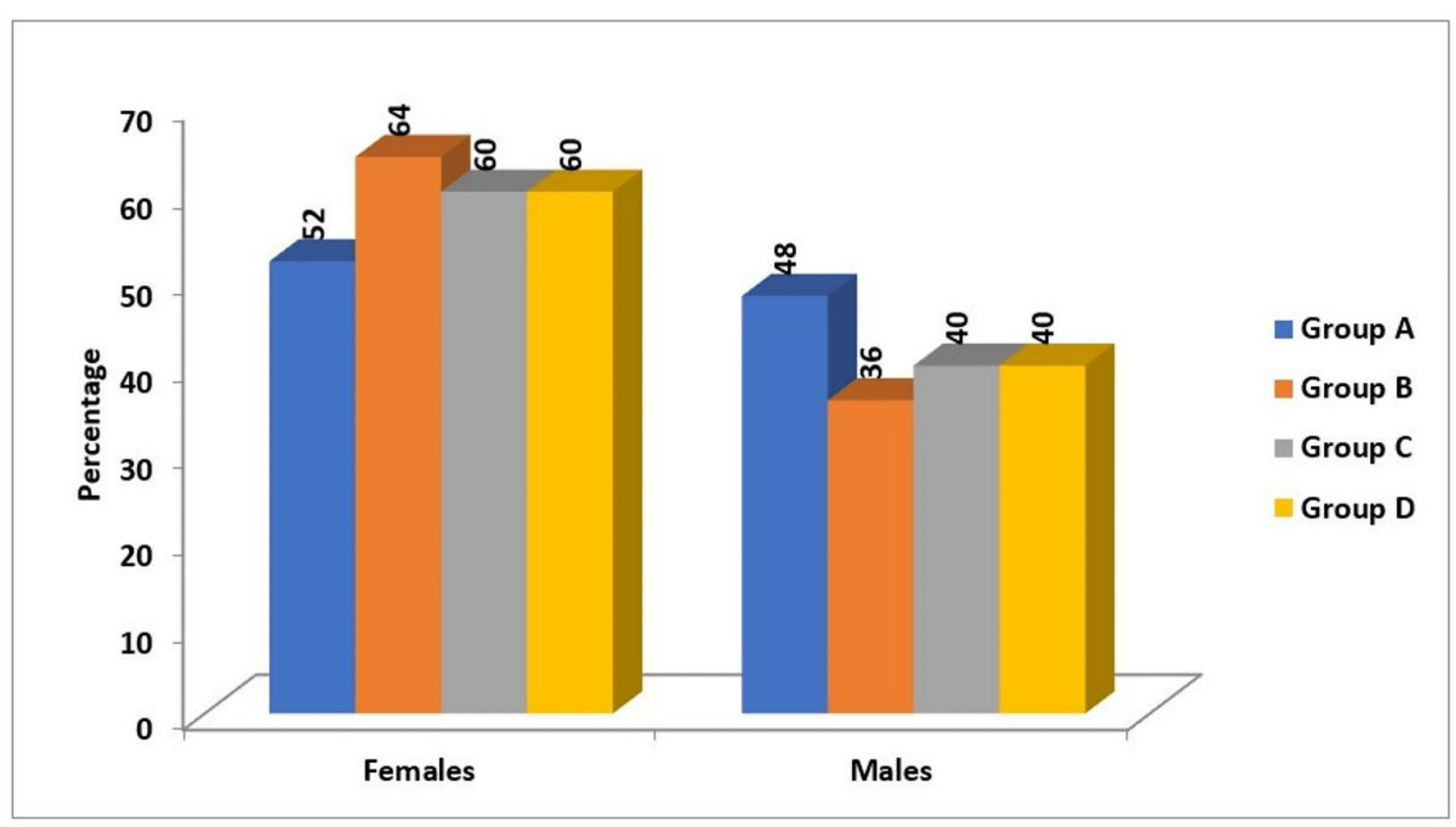
Gender wise distribution of participants.

Among the 100 participants, 59% were females and 41% were males. The distribution of gender across the four study groups is summarized in Figure 2. Group A comprised 13 females (52%) and 12 males (48%). Group B exhibited the highest proportion of female participants, with 16 females (64%) and nine males (36%). In group C, females accounted for 60% (15) and males for 40% (10). Group D included 15 females (60%) and 10 males (40%). The trend indicates a relatively higher usage of earphones among female participants than among their male counterparts.

Distribution of participants based on the duration of earphone usage in past

**Figure 3:**
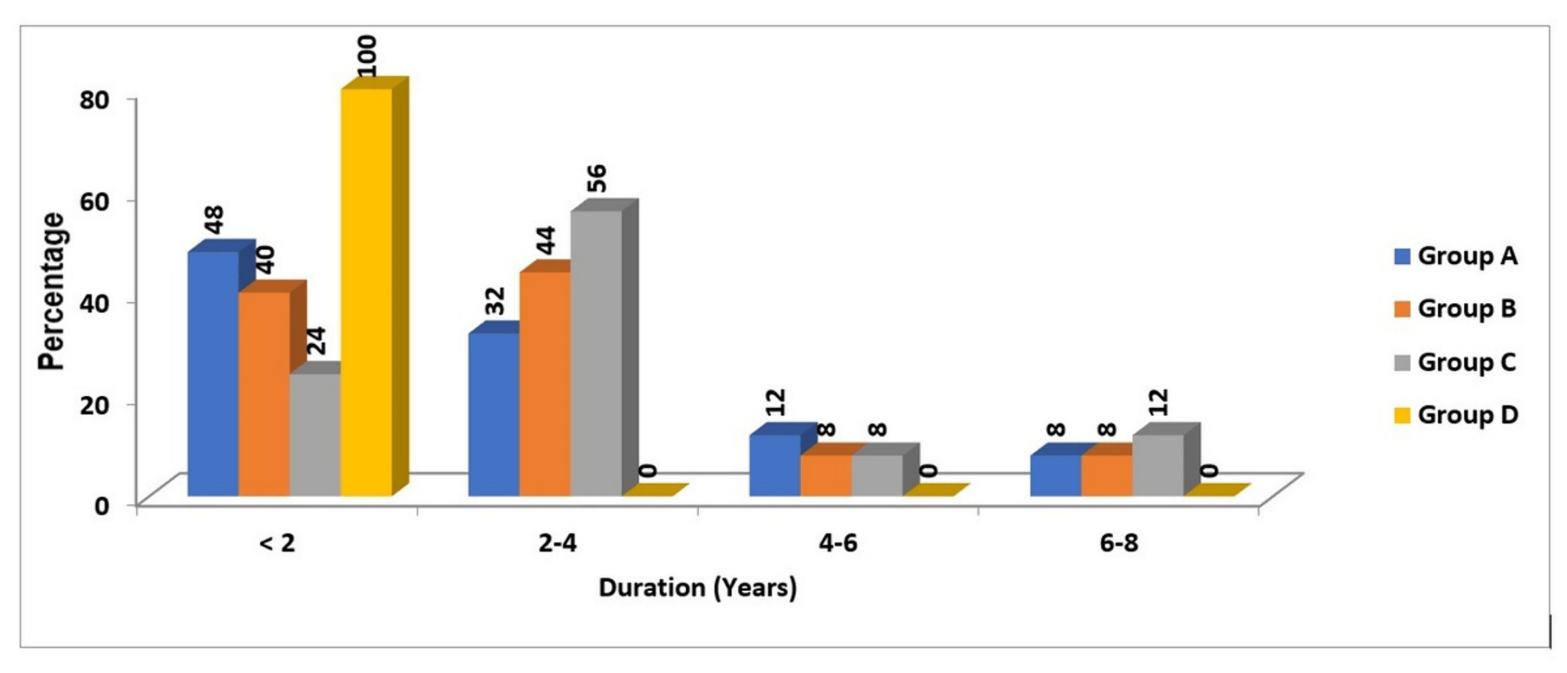
Distribution of participants based on duration of earphone usage in the past.

This study indicated that the majority of participants (n=100) using earphones fall under the category of less than two years (53%), followed by two to four years (33%), four to six years (7%), and six to eight years (7%). In group D, all participants used earphones for less than two years (Figure 3). The mean duration of usage for group A was 2.13±0.36 years, group B was 2.23±0.37 years, and group C was 2.84±0.42 years.

Distribution of participants based on the volume intensity of earphone usage

The participants in each group were subcategorized by listening volume intensity as low (<60%), moderate (60-80%), and high (>80%), based on device volume settings. A majority of participants across the exposure groups reported listening at moderate volume intensity (60-80%). However, high-volume listening (>80%) was most prevalent in group C (40%), followed by group B (28%) and group A (20%), indicating a greater tendency toward potentially harmful listening practices among individuals with prolonged earphone usage duration. In contrast, all participants in the control group (group D) reported low-volume listening (<60%).

**Figure 4:**
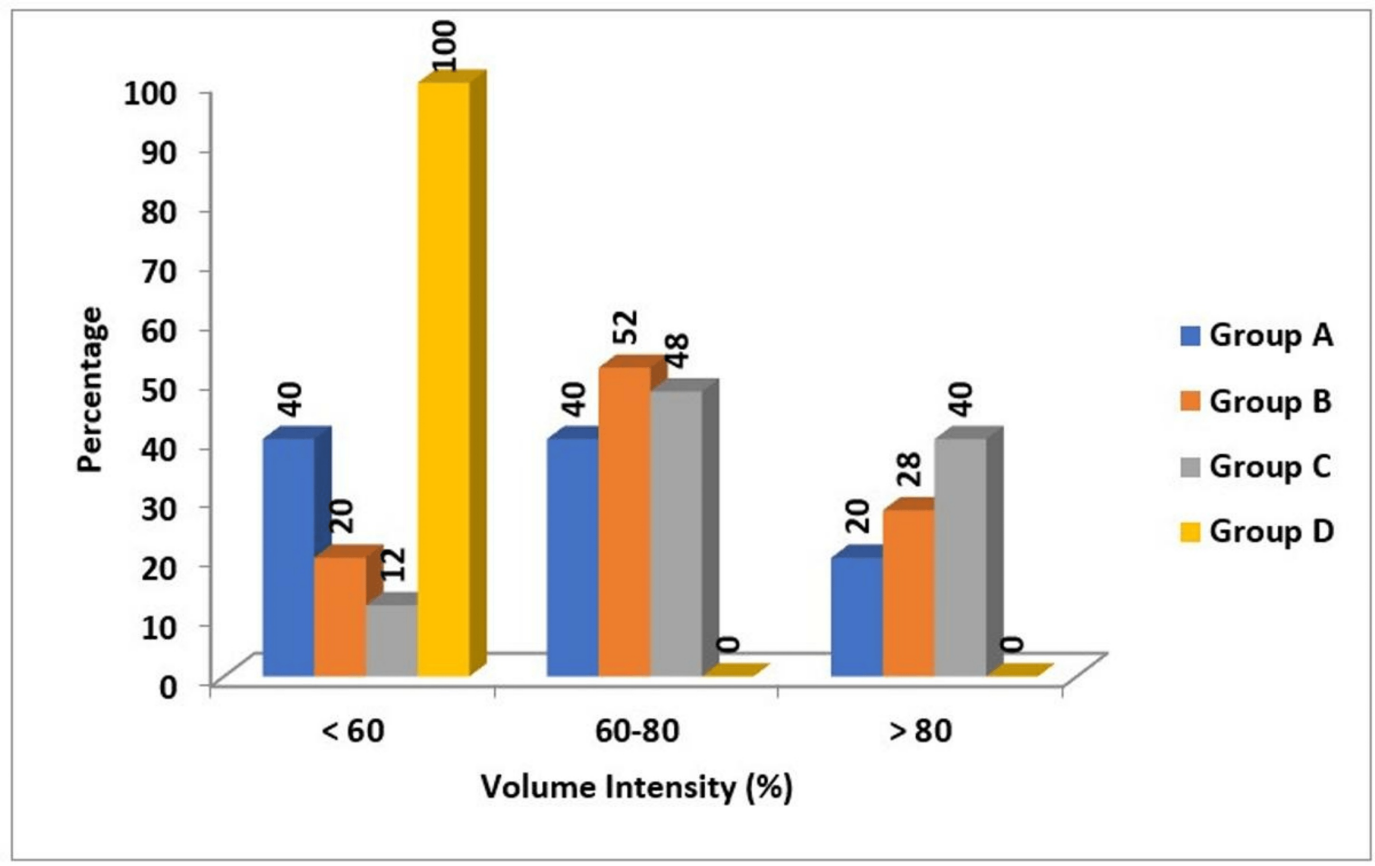
Distribution of participants based on volume intensity.

The findings demonstrated a clear exposure trend, in which prolonged daily earphone usage was associated with a greater proportion of participants reporting higher listening volumes. The higher prevalence of >80% volume exposure in group C corresponds with the greater hearing threshold shifts observed in this group, suggesting a clinically relevant association between listening intensity and early auditory decline. The distribution pattern illustrated in Figure 4 supports the role of both listening duration and volume intensity as important contributory factors in auditory dysfunction among regular earphone users.

Distribution of participants based on otology symptoms 

**Figure 5:**
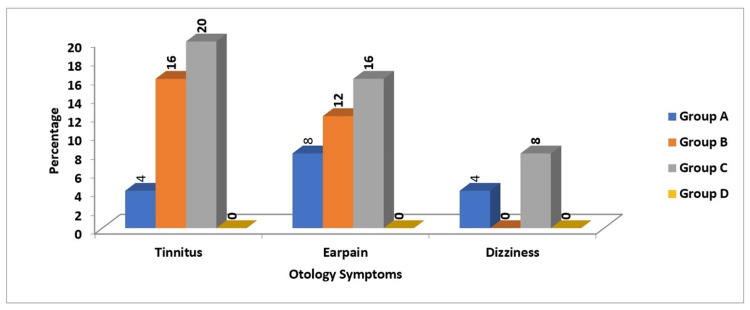
Distribution of participants based on otology symptoms.

The study revealed that 22 participants across the various groups experienced otological symptoms, namely tinnitus (10%), ear pain (9%), and dizziness (3%), potentially associated with earphone use (Figure 5). On the contrary, 78% of participants reported no such complaints. Group C had the highest prevalence, with 20% experiencing tinnitus, followed by group B (16%). Corollary to this, the maximum participants in group C (16%) reported ear pain, followed by group B (12%) and group A (8%). Notably, dizziness was reported by 8% of participants in group C and by 4% in group A. None of the participants experienced any of the otology symptoms in the control group (group D).

The findings indicate that otology symptoms, particularly tinnitus and ear pain, were more prevalent among participants using earphones for longer durations and at higher volumes. This suggests a potential correlation between the extent of earphone exposure and the development of auditory symptoms.

Comparison of mean hearing threshold across study groups over a period of 18 months

**Figure 6:**
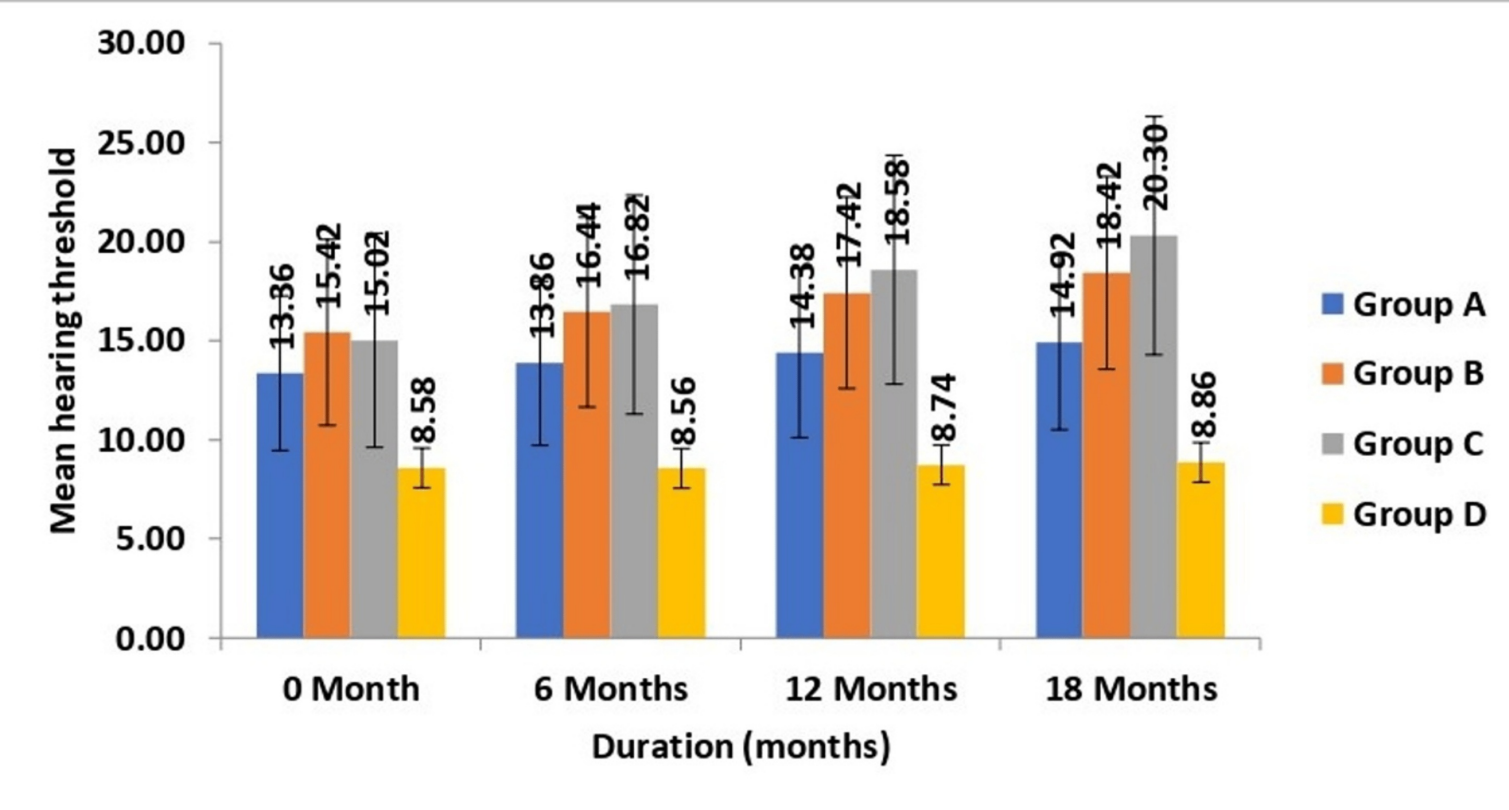
Comparison of mean hearing threshold of groups based on duration of earphones usage.

The mean hearing thresholds of participants across the study groups were analyzed periodically to assess the impact of earphone usage on the potential risk for noise-induced hearing loss. The baseline (0-day) mean hearing threshold varied from 8.58±2.07 dB to 15.42±4.69 dB, indicating a significant difference (F=27.908, p=0.001) across the groups. Group B (15.42±4.69 dB) and group C (15.02±5.40 dB) showed a similar trend; however, a slight decrease was observed in the latter, which may be attributed to individual variability, listening habits, or protective behaviors (Figure 6).

The mean hearing threshold at six months exhibited a substantial increase with increased duration of earphones. The mean threshold for the A-C groups increased from 13.86±4.14 dB to 16.82±5.52 dB, reflecting the cumulative impact of prolonged, high-volume exposure. The statistical analysis revealed a highly significant difference in mean hearing threshold among the groups (F=38.777, p=0.001). 

Additionally, the mean hearing threshold at 12 months in A-C groups exhibited a higher hearing threshold (14.38±4.27 dB to 18.58±5.77 dB) with increasing duration of earphone usage, demonstrating a dose-response relationship. In contrast, group D showed consistent results (8.74±2.16 dB). Group C, with the longest duration of usage, consistently recorded the highest levels of auditory shift, indicating cumulative auditory strain and potential early signs of noise-induced hearing loss. Statistical analysis revealed a highly significant difference among the groups (F=48.677, p=0.001), reinforcing the evidence of a progressive and exposure-dependent increase in hearing thresholds.

At the end of the 18-month follow-up, a similar pattern of progressive increase in hearing threshold (14.92±4.41 dB to 20.30±6.01 dB) was observed across the study groups (A-C). In contrast, group D exhibited a significantly lower mean threshold of 8.86±2.07 dB (Figure 6). Statistical analysis revealed a highly significant difference (p≤0.05) between the groups (F=60.58, p=0.001). The gradual increase in hearing thresholds observed, particularly in groups B and C, may indicate clinically meaningful early auditory changes suggestive of subclinical cochlear stress associated with prolonged and high-volume earphone exposure.

Comparison of mean hearing threshold shift over a period of 18 months

**Figure 7:**
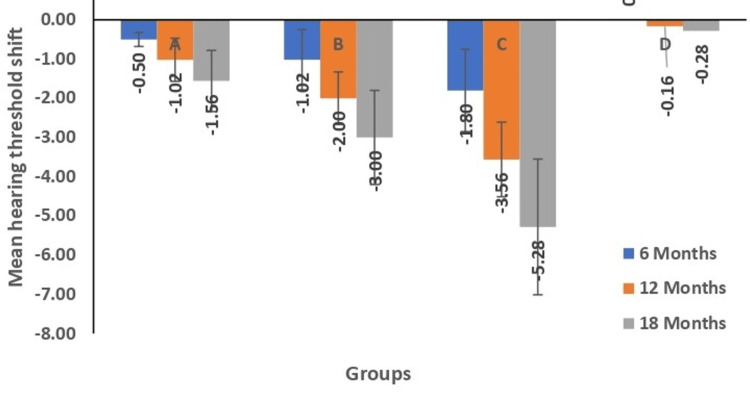
Comparison of mean hearing threshold shift of groups based on duration of earphones usage.

A comparison of mean hearing threshold shifts over a period of 18 months indicated the beginning of measurable auditory fatigue associated with longer exposure to earphones. At six months, the mean shift of group C (-1.80±0.78 dB) was the highest, followed by group B (-1.02±0.55 dB) and group A (-0.50±0.68 dB), while group D (0.02±0.51 dB) exhibited a negligible and non-significant mean hearing threshold shift (Figure 7). The findings suggest that even short-term exposure to moderate-to-high duration earphone use can lead to the initial stage of noise-induced hearing threshold shift. The absence of significant change in the control group further supports that the threshold shifts were likely induced by PLD exposure and not by other environmental or biological factors. Statistical evaluation revealed significant differences in hearing threshold shifts among the groups (F=73.417, p=0.001).

At the 12-month follow-up, a similar trend was observed across the groups; however, a more pronounced decline in hearing threshold shift was observed in group C (-3.56±1.20 dB), reflecting the progressive nature of exposure-related auditory fatigue. Group D recorded a minimal and statistically non-significant mean hearing threshold shift of -0.16±0.62 dB (Figure 7). The findings strongly suggest that prolonged exposure to moderate-to-high volumes via earphones leads to progressive hearing damage over time, with greater deterioration observed in individuals with longer durations. The control group’s near-stable hearing threshold shift further validates that noise exposure from earphones is the primary contributor to the observed hearing threshold shifts. The intergroup differences were found to be highly significant (F=148.908, p=0.001), pointing towards the exposure-dependent relationship between duration of PLD usage and deterioration of hearing threshold shifts.

At 18 months, a substantial and exposure-dependent shift in hearing thresholds was evident among earphone users. Group A recorded a mean hearing threshold shift of -1.56±1.05 dB; group B exhibited a more pronounced shift of -3.00±0.95 dB. Group C experienced the most significant auditory decline, with a mean hearing threshold shift of -5.28±1.73 dB, indicating considerable progressive hearing impairment. In contrast, group D showed a negligible mean hearing threshold shift of -0.28±0.57 dB (Figure 7). The differences in mean hearing threshold shifts across the groups were found to be highly statistically significant (F=172.9, p=0.001), further confirming that the extent of hearing threshold deterioration is strongly related to the duration of earphone usage. The findings thus suggest that prolonged exposure to personal listening devices may contribute to early subclinical auditory dysfunction and noise-induced hearing threshold changes.

Comparison of mean hearing threshold and threshold shift based on duration and volume intensity

**Figure 8:**
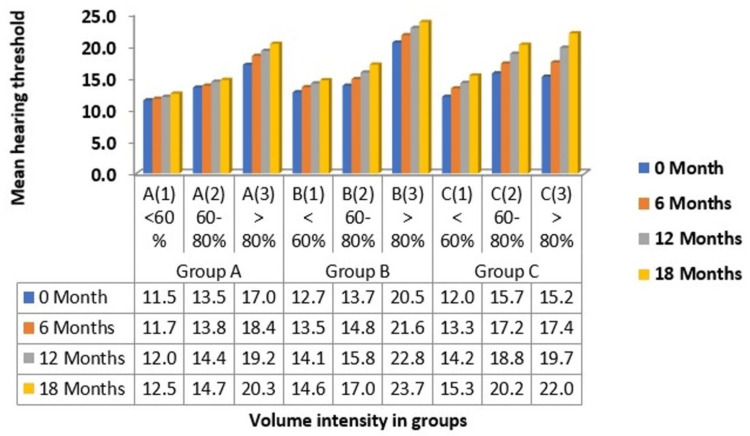
Mean hearing threshold across groups based on duration and volume intensity of earphones usage.

The results revealed that a significant volume-dependent variation in mean hearing thresholds was observed across all time points (baseline, six, 12, and 18 months) in all groups. At baseline, participants in group A using earphones at volumes <60% (A1) had a mean hearing threshold of 11.45±3.35 dB, while those listening at 60-80% (A2) and >80% (A3) volumes had mean hearing thresholds of 13.45±2.89 dB and 17.00±4.32 dB, respectively (F=8.950, p=0.001), indicating higher mean hearing thresholds among those using higher volumes (Figure 8).

**Figure 9:**
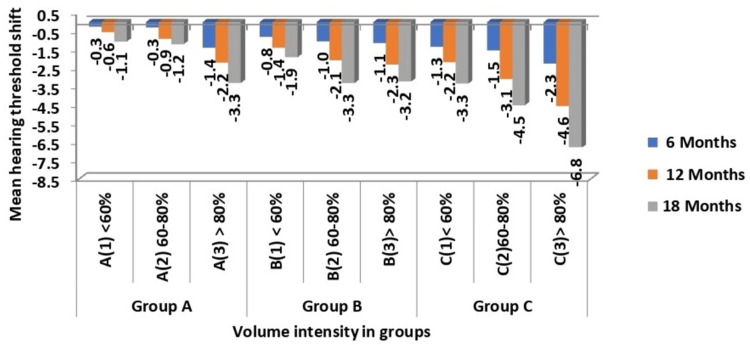
Mean hearing threshold shift across groups based on duration and volume intensity of earphones usage.

The mean hearing threshold shift from baseline further highlighted the progressive damage. By six months, those listening at >80% volume showed a significant shift of -1.40±0.70 dB, compared to -0.30±0.47 dB for 60-80% users and -0.25±0.44 dB for <60% users (F=19.314, p=0.001). The disparity became more pronounced at 12 months for those listening at >80%, with -2.20±0.63 dB in comparison to the users with volume intensity of 60-80% and <60% with -0.90±0.31 dB and -0.55±0.51 dB, respectively (Figure 9). The maximal damage was reported at 18 months with -3.30±0.67 dB for >80%, in comparison to other users listening for lesser volume intensity (-1.20±0.52 dB and -1.05±0.60 dB; F=55.055, p=0.001).

These findings confirm a strong correlation between volume intensity and auditory damage, even among users with shorter daily exposure. The consistent and statistically significant differences (p=0.001 at all intervals) underline the risk of high-volume earphone use, even for moderate durations.

Group B showed a clear and statistically significant difference (F=21.052, p=0.001), indicating that higher volume intensity was associated with higher mean hearing thresholds. At baseline, the mean hearing threshold was 12.70±3.16 dB in participants using earphones at volume intensity <60% (B1); 13.73±2.59 dB for those using 60-80% (B2), and 20.50±4.90 dB for those exceeding 80% volume intensity (B3) (Figure 8).

The differences in mean hearing thresholds across varied groups not only persisted but progressively increased at each follow-up. At six months, mean hearing thresholds increased to 13.50±3.31 dB, 14.77±2.57 dB, and 21.64±5.00 dB (F=21.489, p=0.001). This upward trend continued at 12 months with values of 14.10±3.48 dB, 15.81±2.48 dB, and 22.79±4.81 dB (F=24.234, p=0.001), and further at 18 months reaching 14.60±3.10 dB, 17.04±2.52 dB, and 23.71±5.00 dB (F=24.231, p=0.001). These findings indicate a consistent and statistically significant increase in mean hearing thresholds over time across the users.

Mean hearing threshold shifts showed a progressive increase over time across users. At six months, the shifts for B1 (-0.80±0.63 dB), B2 (-1.04±0.53 dB), and B3 (-1.14±0.53 dB) showed no significant difference (F=1.159, p=0.323). Further, at 12 months, shifts in B1-B3 increased (-1.40±0.52 dB, -2.08±0.63 dB, and -2.29±0.61 dB, respectively) and were statistically significant (F=6.728, p=0.003). At 18 months, the decline was more pronounced and statistically significant (-1.90±0.57 dB, -3.31±0.84 dB, and -3.21±0.80 dB; F=12.408, p=0.001), indicating a clear time and exposure-dependent decline in mean hearing threshold shift (Figure 9).

The findings indicate a progressive increase in mean hearing threshold and hearing loss over time, with more pronounced effects at higher volumes. Although the results at six months in mean hearing threshold shift were not significant, the cumulative effects of both volume intensity and duration became evident at 12 and 18 months, emphasizing the risk of developing NIHL.

Group C exhibited a clear time and exposure-dependent trend in hearing outcomes. Mean hearing thresholds showed a gradual increase from baseline to 18 months across all volume intensity categories (<60%, 60-80%, >80%), with higher values consistently observed at higher volume listening intensities (Figure 8). Although inter-group differences in mean thresholds were not statistically significant at any time point, a progressive upward trend was evident and more pronounced in higher volume intensity users.

In contrast, mean hearing threshold shifts demonstrated significant and cumulative deterioration over time. At six months, minimal variation in mean hearing threshold shifts was observed across C1, C2, and C3; however, the differences became statistically significant and progressively intensified at 12 and 18 months, with the greatest decline noted in the >80% volume intensity group (C3) (Figure 9). The magnitude of shift increased consistently with both duration and volume of exposure, with statistically significant differences across all follow-ups.

Thus, the findings indicate that prolonged and high-volume use of PLDs leads to progressive auditory losses. While early changes in mean hearing thresholds may appear subtle, cumulative threshold shifts provide strong evidence of duration and volume intensity-dependent auditory damage, suggesting an increased risk of noise-induced hearing loss with sustained exposure.

The findings indicate a dose-dependent relationship, where both the duration and intensity of earphone use contribute to gradual hearing deterioration. Group C, with the longest exposure, experienced the most substantial hearing shift over time, followed by group B and then group A. Meanwhile, group D maintained near-normal hearing thresholds, reinforcing the fact that reduced exposure to PLDs helps preserve auditory health.

The progressive increase in hearing thresholds over 18 months points towards the cumulative auditory impact of long-term and high-volume earphone usage among young adults. The results highlight the need for early intervention, public education, and promotion of safe listening habits to mitigate the risk of noise-induced hearing loss.

## Discussion

Noise-induced hearing loss is a prevalent yet largely preventable auditory condition that results from prolonged or repeated exposure to loud sounds. Traditionally associated with occupational and recreational noise exposure, NIHL has become increasingly common in non-occupational environments, particularly with the widespread use of PLDs such as in-ear earphones. These devices deliver sound directly into the ear canal and, when used at high volumes for extended durations, can lead to temporary or permanent hearing threshold shifts by damaging the sensory hair cells in the organ of Corti [5].

With the ubiquity of smartphones and PLDs in daily life, especially among adolescents and young adults, the risk of NIHL has become more widespread. Various studies have indicated that both the duration of exposure and volume intensity are critical factors influencing the onset and progression of hearing loss [6,9]. Continuous exposure to loud sounds has been associated with increased metabolic activity in cochlear cells, leading to oxidative stress [10], mitochondrial dysfunction [11], glutamate excitotoxicity [12], and activation of inflammatory and apoptotic pathways, which contribute to auditory cell damage and eventual sensorineural hearing loss [13,14].

This prospective study aimed to evaluate the impact of the use of in-ear earphones on mean hearing thresholds in young adults over a period of 18 months. The study enrolled 100 participants following the inclusion and exclusion criteria in the age group of 18-25 years. The selected participants were categorized into four groups based on the duration of daily usage of in-ear earphones. The groups were classified as follows: group A (1-2 h/day); group B (2-4 h/day); group C (>4 h/day), and group D (≤1 h or no earphone use), which served as control. Each group's participants were further subcategorized as per volume intensity as low (<60%) (A1, A2, A3), moderate (60-80%) (B1, B2, and B3), and high (>80%) (C1, C2, C3) volume levels.

Demographics and otology symptoms

The majority of earphone users were between 21 and 22 years old, consistent with previous studies such as Ahmed et al. and You et al., which reported high PLDs usage among college students [15,16]. In this study, 59% of users were female and 41% were male, aligning with findings from the study by Ansari et al., who conducted a cross-sectional study on the prevalence and patterns of earphone and music player use among adolescents [17]. The study showed a history of hearing problems among adolescents (44.3%), with a higher percentage in females (47%) than in males (42.2%). On the contrary, another study conducted on Korean college students (20-30 years) showed similar usage irrespective of gender (50.1% and 49.9% for males and females, respectively) [16].

In our study, otological symptoms such as tinnitus (10%), ear pain (9%), and dizziness (3%) were reported, predominantly among groups with higher earphone usage. Tinnitus was most common among those listening at high-volume intensity and for longer durations. The findings align with Rasouli et al., who identified that 37.63% (5.37% conductive hearing loss {HL}: 29.03% sensori-neural HL and 3.22% mixed HL) of the total (n=93) adults were suffering from tinnitus [18]. The study emphasized the need for comprehensive hearing healthcare services. The low incidence of dizziness may be attributed to the young age (18-21 years) of the respondents, who may lack the comorbid manifestations of vestibular conditions generally seen in older populations. The results are supported by studies conducted by Rasouli et al. and Fatima et al., wherein the older participants suffered more from dizziness or vertigo attributed to compromised vestibular function than their younger counterparts [18,19]. The former study reported that 13.97% of the total patients (n=93) suffered from vertigo. The vertigo was more pronounced among patients with sensorineural hearing loss (SNHL) (8.60%), followed by patients with mixed hearing loss (3.22%) and conductive hearing loss (2.15%). On the other hand, the latter study reported that the usage of earphones for 7-9 h among call center operators showed symptoms of vertigo. The study further emphasized that multiple factors, such as age, working hours, and years of work experience among the users, may lead to vertigo or dizziness.

Hearing threshold changes based on the duration of earphone usage

The results demonstrated a progressive and statistically significant (p≤0.05) increase in mean hearing thresholds over the 18-month follow-up period among participants who used in-ear earphones. The magnitude of hearing threshold shift increased in a dose-dependent manner, with group C (>4 h/day) showing the greatest auditory deterioration, followed by groups B and A. Even participants with lower exposure levels exhibited early subclinical auditory changes, whereas the control group (group D) showed minimal variation and relative auditory stability. The findings indicate that the duration of earphone usage is an important modifiable risk factor contributing to early auditory dysfunction.

Although the observed changes remain within mild clinical limits, they may represent early indicators of noise-induced hearing loss and could progress if unsafe listening practices persist. The findings are consistent with a previous study conducted by Murray et al., which reported that hearing threshold shifts depend on exposure duration, intensity, and individual susceptibility [20].

Hearing threshold shift based on the duration of earphone usage

The mean hearing threshold shift across groups A, B, and C showed a significant progressive increase, with group C exhibiting the most pronounced shifts (1.80-5.28 dB) over the study period. In contrast, the control group showed minimal threshold shifts across all time points, ranging from 0.02 to 0.28 dB. The findings are consistent with prior research documenting significant hearing threshold shifts in individuals with prolonged PLD usage, particularly at higher exposure levels [21]. Similarly, Gottfriedová et al. reported that higher hearing threshold changes can be seen even in young otologically healthy adults at conventional frequencies (CF) owing to daily use, increased duration, and loud volume [22]. The authors further emphasized that daily use of PLDs for extended duration at higher volumes resulted in greater hearing losses at certain frequencies.

Mean hearing threshold shift across groups based on volume intensity

The participants within the A, B, and C groups using earphones at higher volumes experienced greater hearing threshold shifts. For instance, in group C, those listening at >80% volume (C3) showed hearing threshold shifts of 2.25 dB, 4.55 dB, and 6.80 dB at six, 12, and 18 months, respectively. In comparison, those in the <60% group (C1) showed lower (1.33 dB, 2.17 dB, and 3.33 dB at six, 12, and 18 months, respectively) but still increasing trend in volume intensity threshold shifts over the respective time intervals.

The results are in accordance with a previous study conducted by Engdahl and Aarhus, wherein the association was clearly established, demonstrating that listening at higher volume intensities increased the hearing threshold [21]. These studies correlate well with the progression observed in our higher-exposure subgroups.

Conversely, users in the low-volume categories experienced minimal mean shifts in hearing threshold. The results are supported by the findings of studies conducted by Paping et al., which reported that mild-to-moderate volume use, especially with breaks or under quiet environmental conditions, may not cause significant long-term auditory damage in adolescents [23].

Comparative mean hearing threshold and its shift across groups based on duration and volume intensity of earphone usage

The results affirm that both prolonged duration and high-volume intensity are independent yet interacting risk factors for hearing threshold deterioration. Participants in group C who used earphones for over 4 h daily at >80% volume (C3) showed the greatest auditory threshold shifts, highlighting the cumulative effects of exposure duration and intensity. The volume-duration interaction aligns with findings by Sulaiman et al., who reported significant and often irreversible damage under similar conditions [24]. Thus, the study highlights the need for early intervention, public education, and promotion of safe listening habits to mitigate the risk of hearing threshold deterioration.

The present study, although comprehensive, had certain limitations that should be acknowledged while interpreting the findings. The sample size was relatively small and restricted to a narrow age group (18-25 years), which may limit the applicability of the findings to broader and more heterogeneous populations. In addition, the participants were recruited from a single setting, which may further restrict the generalizability of the observations across different demographic and socioeconomic backgrounds.

Although the 18-month follow-up period was adequate to identify early auditory alterations, it may not have been sufficient to determine the long-term progression, reversibility, or permanence of hearing impairment associated with prolonged earphone usage. The study primarily relied on conventional pure-tone audiometry for auditory assessment. While PTA remains a standard clinical tool, the absence of advanced audiological investigations, such as extended high-frequency audiometry and otoacoustic emission (OAE) testing, may have limited the detection of subtle or early subclinical cochlear dysfunction.

Furthermore, despite efforts to minimize bias through screening and exclusion criteria, certain confounding variables, including environmental and recreational noise exposure, gaming habits, use of multiple audio devices, lifestyle-related listening practices, and inter-individual variability in susceptibility to noise-induced hearing damage, could not be completely controlled. The assessment of listening volume was also based on self-reported device volume settings rather than objectively measured sound intensity levels in decibels, which may have introduced variability in exposure estimation.

Despite these limitations, the study provides important longitudinal evidence suggesting that prolonged, high-volume earphone use may contribute to progressive auditory threshold shifts, even in young adults. The findings highlight the importance of safe listening practices, early auditory screening, and increased awareness regarding recreational noise exposure. Future large-scale multicentric studies incorporating objective audiological assessments and longer follow-up durations are warranted to better understand the long-term auditory consequences of personal listening device usage and to support the development of evidence-based preventive strategies.

## Conclusions

This prospective study demonstrated that extended usage of earphones among young adults is associated with hearing threshold shifts. The observed changes were positively correlated with both duration and volume intensity of usage, identifying these factors as significant and modifiable determinants of early auditory decline. Participants with higher exposure, particularly those using in-ear earphones for more than 4 h per day at volumes exceeding 80%, demonstrated a higher degree of hearing threshold shift. In contrast, the control group, with minimal or no earphone usage, showed no notable shifts in hearing thresholds, highlighting the potential auditory risks associated with regular earphone usage.

Although the magnitude of threshold shifts remained subtle, they may represent early indicators of noise-induced hearing loss and potential cumulative cochlear damage with continued unsafe listening practices. These findings highlight the need for targeted public health interventions promoting safe listening behaviors, including limiting daily usage duration to less than 1 h, maintaining volume levels below 60%, and practicing regular auditory rest periods. This study also emphasizes the importance of early detection and routine hearing evaluations for frequent earphone users. Future research, especially studies involving larger populations for longer duration and advanced audiological assessments such as otoacoustic emission testing, will be crucial in deepening our understanding of the long-term consequences of earphone usage and in guiding the development of effective preventive measures.

## Figures and Tables

**Figure 10:**
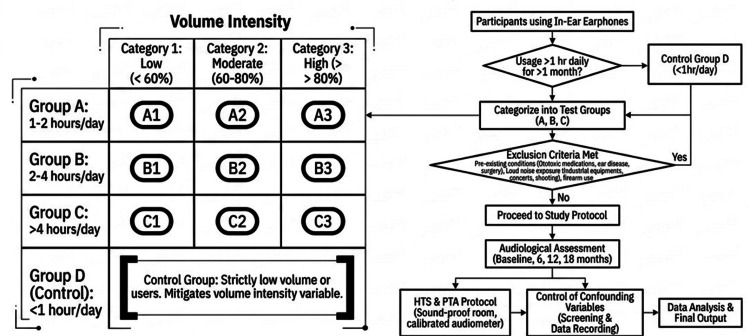
Schematic representation of study. HTS: hearing threshold shift; PTA: pure-tone audiometry

## Appendices

**Table 1:** Structured questionnaire used for assessing earphone usage patterns, listening habits, noise exposure, and self-reported auditory symptoms among study participants.

S. no.	Questions	Categories		0 month	6 months	12 months	18 months
1	Do you use earphones?	Yes, no					
2	If yes, are your earphones single-sided/dual-sided?	Single, dual					
3	If single, which ear do you use most often?	Right, left, not sure					
4	What devices are your earphones most commonly connected to?	Computer, mobile, MP3 player					
5	How long have you been using earphones?	<1 month, >1 month					
6	If more than 1 month, specify the duration (in years)						
7	What is the duration you use earphones on an average per day?	<1 h, 1-2 h, 2-4 h, >4 h					
8	When you use earphones, what volume do you use most often?	<60%, 60-80%, >80%					
9	What is your favorite place to wear earphones?	Gym, driving, street, home, others					
10	When using earphones, do you pause to rest your ears?	Always, most of the time, sometimes, very little, never					
11	What kind of headphones do you usually use?	In-ear earphones, overhead, headphones					
12	How do you rate your hearing?	No hearing problem, mild hearing problem, moderate hearing problem, severe hearing problem					
13	Have you ever been exposed to significant noise in your life?	Yes, no					
14	Have you ever taken any medication that you were told may affect your hearing?	Yes, no					
15	Have you had any sudden or rapid changes in your hearing (within 90 days)?	Yes, no					
16	Do you have itching in your ears?	Yes, no					
17	Do you experience any dizziness?	Yes, no					
18	Do you get pain in your ears?	Yes, no					
19	Do you get any infections or discharge from your ears (not including wax)?	Yes, no					
20	Do you hear any rushing, hissing, ringing, beating, pulsing, or any other noises in your ears, often called tinnitus?	Yes, no					
21	Do you have hearing difficulty during conversation in a group with no background noise?	Yes, no					
22	Has anyone in your family had a hearing loss not associated with aging?	Yes, no					
23	Do you have hearing difficulty when hearing the television or radio at normal volume?	Yes, no					
24	Do you have difficulty hearing the phone ring from another room?	Yes, no					
25	Do you have difficulty hearing the doorbell or knocker?	Yes, no					
